# HPV type-related chromosomal profiles in high-grade cervical intraepithelial neoplasia

**DOI:** 10.1186/1471-2407-12-36

**Published:** 2012-01-24

**Authors:** Mariska Bierkens, Saskia M Wilting, Wessel N van Wieringen, Mark A van de Wiel, Bauke Ylstra, Chris JLM Meijer, Peter JF Snijders, Renske DM Steenbergen

**Affiliations:** 1 Department of Pathology, Unit of Molecular Pathology, VU University Medical Center, PO box 7057, 1007 MB Amsterdam, The Netherlands; 2 Epidemiology & Biostatistics, VU University Medical Center, De Boelelaan 1118, 1081 HV Amsterdam, The Netherlands; 3 Department of Mathematics, VU University, de Boelelaan 1081A, 1081 HV Amsterdam, The Netherlands

**Keywords:** Array CGH, Cervical cancer, Chromosomal aberrations, High-grade cervical intraepithelial neoplasia, HPV

## Abstract

**Background:**

The development of cervical cancer and its high-grade precursor lesions (Cervical Intraepithelial Neoplasia grade 2/3 [CIN2/3]) result from a persistent infection with high-risk human papillomavirus (hrHPV) types and the accumulation of (epi)genetic host cell aberrations. Epidemiological studies have demonstrated variable CIN2/3 and cancer risks between different hrHPV types. Recent genomic profiling studies revealed substantial heterogeneity in the chromosomal aberrations detected in morphologically indistinguishable CIN2/3 suggestive of varying cancer risk. The current study aimed to investigate whether CIN2/3 with different hrHPV types vary with respect to their chromosomal profiles, both in terms of the number of aberrations and chromosomal loci affected.

**Methods:**

Chromosomal profiles were determined of 43 p16^INK4a^-immunopositive CIN2/3 of women with long-term hrHPV infection (≥ 5 years). Sixteen lesions harboured HPV16, 3 HPV18, 14 HPV31, 1 HPV33, 4 HPV45, 1 HPV51, 2 HPV52 and 2 HPV58.

**Results:**

Unsupervised hierarchical clustering analysis of the chromosomal profiles revealed two major clusters, characterised by either few or multiple chromosomal aberrations, respectively. A majority of 87.5% of lesions with HPV16 were in the cluster with relatively few aberrations, whereas no such unbalanced distribution was seen for lesions harbouring other hrHPV types. Analysis of the two most prevalent types (HPV16 and HPV31) in this data set revealed a three-fold increase in the number of losses in lesions with HPV31 compared to HPV16-positive lesions. In particular, losses at chromosomes 2q, 4p, 4q, 6p, 6q, 8q & 17p and gain at 1p & 1q were significantly more frequent in HPV31-positive lesions (FDR < 0.2).

**Conclusions:**

Chromosomal aberrations in CIN2/3 are at least in part related to the hrHPV type present. The relatively low number of chromosomal aberrations observed in HPV16-positive CIN2/3 suggests that the development of these lesions is less dependent on genetic insult than those caused by other types like HPV31.

## Background

Persistent infection with mucosal high-risk human papillomaviruses (hrHPVs) has been causally related to the development of cervical cancer [1]. HPV types can be grouped into genera (α, β, γ, μ, η) with types belonging to the same genus generally sharing common characteristics, such as tissue tropism and oncogenic potential [2]. The hrHPV types encompass the α5, α6, α7, α9 and α11 species of the α-genus. There are around 13 hrHPV types, of which types 16 (α9 species) and 18 (α7 species) are the cause of approximately 70% of all cervical cancers [3]. Though infection with hrHPV is common, the majority of infections are cleared by the immune system. Only in some instances pre-cancerous lesions arise, so-called cervical intraepithelial neoplasia (CIN) [4]. Not all CIN represent direct precursor stages of cervical cancer. Low-grade CIN (CIN1) mostly reflect productive hrHPV infections, in which active viral replication and virion production are strongly related to the differentiation programme of the infected epithelium [5]. High-grade CIN (CIN2/3) usually harbour transforming infections, characterised by deregulated expression of viral oncogenes *E6 *and *E7 *in proliferating cells [5]. These lesions have the potential of malignant progression towards invasive carcinoma, largely due to the inactivation of tumour suppressors p53 and pRb by viral oncoproteins. This results in the accumulation of specific (epi)genetic changes in the host cell genome that may further drive the progression to a malignant phenotype [1,6]. One of the features of a transforming infection is over-expression of p16^INK4a ^due to deregulated E7 expression, making p16^INK4a ^a suitable marker to distinguish cervical pre-cancer from productive viral infections [7-10].

CIN2/3 are believed to represent a heterogeneous disease. While these may be rapidly induced within 2-3 years following hrHPV infection, progression to invasive cervical carcinoma may still take another 10-30 years [5,11,12]. The heterogeneous nature is substantiated by the fact that some features common to cervical carcinomas are only found in a subset of CIN2/3. These include for instance up-regulated hTERT, VEGF, c-fms and COX-2 expression, but also methylation-mediated silencing of (candidate) tumour suppressor genes such as *CADM1*, *MAL*, *CALCA*, *RARβ2*, *TFPI2*, *SPARC*, *CCNA *and *hsa-miR-124 *[13-20]. Our previous studies demonstrated that heterogeneity also exists at the chromosomal level [21]. Comparative genomic hybridisation microarray (arrayCGH) analysis of p16^INK4a^-positive CIN2/3 demonstrated two subsets, one showing few chromosomal aberrations and the second subset containing multiple aberrations akin to those found in cervical carcinomas [21]. We found that this heterogeneity is, at least in part, related to varying duration of lesion existence, as was approximated by the duration of preceding hrHPV infection [6]. CIN3 with a long-term HPV infection (≥ 5 years) had a significantly higher number of chromosomal aberrations compared to CIN3 with a short-term HPV infection (< 5 years).

There is now compelling evidence that different hrHPV types confer variable risks of CIN2/3 and cervical cancer. This likely reflects various viral properties that become manifest at different stages during cervical cancer development. Firstly, the risk of developing CIN2/3 is strongly coupled to type-specific differences in viral persistence, most likely reflecting different efficiencies in evading the immune system [22,23]. Secondly, carcinogenic properties seem to vary between different hrHPV types, as reflected by their diverse prevalence in CIN2/3 versus cervical cancer [24-31]. Particularly HPV types 16 and 18 predominate in cervical cancers, whereas other types, like 31 and 33 are relatively more prevalent in CIN2/3 [24-31]. To what extent these differences in transforming properties are reflected by type-specific differences in chromosomal aberrations is still unknown.

The aim of the current study was to investigate whether CIN2/3 containing different hrHPV types also differ with respect to their chromosomal signatures, both at the numerical level (i.e. the number of aberrations) and structural level (i.e. the specific loci affected). Since our previous study showed that the difference in number of aberrations was correlated to duration of preceding hrHPV infection, we only included cases with more than 5 years preceding hrHPV infection in order to minimise bias due to duration of existence. In addition to comparison of HPV16-positive lesions to those harbouring all other high-risk types (HPV_non16_) and HPV31-positive cases, also, CIN2/3 with HPV types from the α9 species (HPVα9, types 16, 31, 33, 52, 58) were compared to those with α5&α7 species combined (HPVα5&α7, types 18, 45, 51). The latter branches are located in close proximity to each other in the phylogenetic tree of HPV types and are clearly separated from the α9 species [32].

## Methods

### Tissue specimens

Formalin-fixed paraffin embedded CIN2/3 biopsies (4 CIN2, 29 CIN3) of 43 women who participated in the population based screening study Amsterdam (POBASCAM) were used in this study [33]. A subset (n = 24) of these lesions has also been described previously [6]. Lesions were detected in the second screening round of the POBASCAM trial in women who at baseline, 5 years earlier, had an hrHPV-positive smear with normal cytology. The lesions contained the same hrHPV type present in the baseline smear, as determined using the GP5+/6+ RLB system [34]. Sixteen lesions harboured HPV16 (α9), 3 HPV18 (α7), 14 HPV31 (α9), 1 HPV33 (α9), 4 HPV45 (α7), 1 HPV51 (α5), 2 HPV52 (α9) and 2 HPV58 (α9). With exception of two cases the lesions only harboured one hrHPV type. In case of lesion number HPV16.12, HPV16 was the type detected at both baseline and in the biopsy. However, the biopsy contained HPV51 as well. In case of lesion number HPV18.1, HPV18 was detected at both baseline and in the biopsy, the latter contained HPV16 as well. Given the history of HPV type persistence, these lesions were classified as being caused by HPV16 and HPV18, respectively. Immunohistochemical staining for p16^INK4a ^was performed using p16^INK4a ^Ab-4, Clone 16P04 (Lab Vision Corporation, Neomarkers, Fremont, California, USA). All lesions showed diffuse staining for p16^INK4a ^in at least the lower 2/3 of the epithelium, indicating the presence of a transforming infection. Histological review of lesions was performed by two experienced pathologists (dr. F.J.van Kemenade and dr. M.C.G Bleeker). The average age of the women was 41, ranging from 34-56 years. There was no significant difference in the average age of the women diagnosed with lesions containing HPV16 compared to those containing HPV31 or other types of hrHPV. This study was approved by the Institutional Review Board of the VU University Medical Center.

### ArrayCGH of microdissected tissues

Microdissection of dysplastic areas of the biopsies, DNA extraction, amplification, labeling and across arrayCGH hybridisation to the 2×105 K arrayCGH platform (Agilent Technologies, Palo Alto, USA) as well as hierarchical clustering was performed as described previously [6]. ArrayCGH analysis of a subset (n = 24) of lesions included in the present study was also performed in this previous study. Array data is available from the Gene Expression Omnibus (GEO, http://www.ncbi.nlm.nih.gov/projects/geo/) through series accession number GSE31241.

### Statistical analysis

Cluster assignment of lesions with HPV16 and those with types other than HPV16 (HPV_non16_) was compared using the two-tailed Fisher exact-test. The non-parametric Mann-Whitney-*U *test was used for comparisons of the proportion of altered features (percentage of oligonucleotides deviating from the normal state) between lesions with HPV16 versus those with HPV_non16 _or HPV31, as well as comparisons between lesions with hrHPV types from the α9 species (HPVα9) versus those from the α5 and α7 species combined (HPVα5&α7). The χ^2^-test (CGHtest_version_1.1) was used to determine whether there was an association between chromosomal aberration patterns of CIN2/3 and hrHPV type [6,35]. The test procedure includes a permutation-based false discovery rate (FDR) correction for multiple testing. Chromosomal profiles with < 1.5% altered probes in total were excluded from this test in order to only investigate regions that were differentially affected. It should be noted that the lesions excluded from this analysis were equally distributed over the HPV types present. Differences were considered significant if the false discovery rate (FDR) was < 0.2. Pathway analysis was performed on all genes located within the significantly different chromosomal regions using Ingenuity Pathway Analysis version 8.7, build 99759; content version 3203, build ing_merak (Ingenuity Systems, Redwood City, California).

## Results

### Clustering analysis displays the heterogeneity of CIN2/3

**Figure 1:**
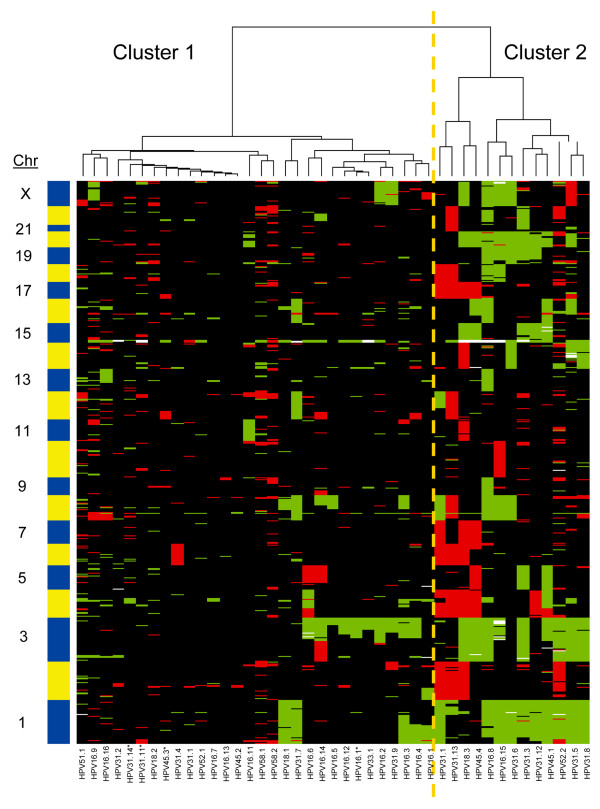
**Results of WECCA clustering on all the CIN2/3**. Cluster 1 contains CIN2/3 with few chromosomal aberrations; the majority of the samples in the right subcluster primarily show a gain of 3q. Samples in cluster 2 have more chromosomal aberrations, including gained regions on chromosomes 1, 3q and 20 and more chromosomal losses. Samples with * are CIN2, the others CIN3.

**Figure 2:**
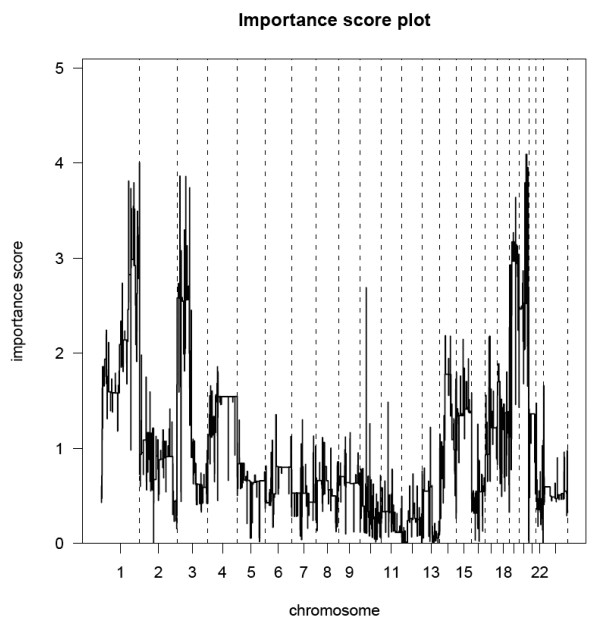
**Importance score plot between lesions in cluster 1 versus lesions in cluster 2**. For each chromosomal region the maximum pair wise symmetrised Kullback-Leibler divergence was determined. Gains of chromosomal regions 1q, 3p and 20q are revealed as the most striking differences of the lesions the two clusters.

Chromosomal profiles of 43 p16^INK4a^-positive, microdissected CIN2/3 were determined using high-resolution arrayCGH. Unsupervised hierarchical clustering analysis was performed to compare these profiles in an unbiased manner as a first investigation into a possible association of aberration pattern with hrHPV type. Two main clusters were identified (Figure 1). Cluster 1 contained 30 samples with few aberrations, including 14 cases with HPV16, 2 with HPV18, 7 with HPV31, 1 with HPV33, 2 with HPV45, 1 with HPV51, 1 with HPV52 and 2 with HPV58. Cluster 2 contained 13 samples, including 2 cases with HPV16, 1 with HPV18, 7 with HPV31, 2 with HPV45 and 1 with HPV52. The difference in distribution of lesions with HPV16 compared to HPV_non16 _over clusters 1 and 2 was borderline significant (*p *= 0.086), with 87.5% of lesions with HPV16 belonging to cluster 1. Lesions with HPV31 were equally distributed over the clusters. Comparison of the distribution of other hrHPV types individually was not possible due to the small numbers of each of these individual types. The major differences in chromosomal aberrations occurring in lesions of clusters 1 and 2 were determined by the maximum pair-wise symmetrised Kullback-Leibler divergence. In Figure 2 the importance scores are shown per chromosomal region, with a higher score indicating a larger contribution of the related aberration to the clustering results. This revealed gains of chromosomal regions on 1q, 3p and 20q to be most discriminatory between the clusters. Yet, within cluster 1 a subgroup of 13 lesions could be discerned that are characterised by gains of chromosome 3q and/or 1. Interestingly, the majority of these lesions (n = 9) harboured HPV16.

### CIN2/3 with HPV16 have fewer and different chromosomal aberrations than those with other hrHPV types

The average percentage of all the microarray oligonucleotides that deviated from the normal state (a proxy for the number of aberrations) occurring in the CIN2/3 was determined and compared between lesions with different hrHPV types (Table 1). The percentage of aberrant oligonucleotides for lesions with HPV16 was 11.4%, for lesions with HPV_non16 _16.1% and those with HPV31 18.3%. The percentage of aberrant oligonucleotides in lesions with hrHPV types from the α9 species (HPVα9) was 13.6% and 17.8% for lesions with hrHPV types from the α5 and α7 species combined (HPVα5&α7). Whereas the percentage of total aberrations, or losses and gains separately, was not significantly different amongst these groups, lesions with HPV_non16 _and HPV31 specifically displayed an approximately three-fold increase in the percentage of losses compared to lesions with HPV16. Comparison of lesions with HPVα9 versus HPVα5&α7 revealed a two-fold increase in the number of losses in lesions with HPVα5&α7.

**Table 1:** The percentage of aberrant probes in lesions with different hrHPV types

	Losses%		Gains%		Total%	
	average	range	average	range	average	range
HPV16 (n = 16)	2.19%	0.005-9.82%	9.18%	0.028-43.33%	11.37%	0.19-43.91%
HPV_non16 _(n = 27)	6.65%	0.015-37.75%	9.49%	0.015-37.75%	16.14%	0.12-54.04%
HPV31 (n = 14)	6.67%	0.015-37.75%	11.64%	0.081-31.40%	18.31%	0.20-46.46%
HPVα9 (n = 35)	4.20%	0.005-37.75%	9.38%	0.028-43.33%	13.58%	0.19-46.46%
HPVα5+α7 (n = 8)	8.45%	0.093-36.60%	9.36%	0.025-30.18%	17.80%	0.12-54.04%

Shown are the average percentage and the range of deviating array oligonucleotides. An increased amount of aberrations can be seen in the lesions with types other than HPV16

**Figure 3:**
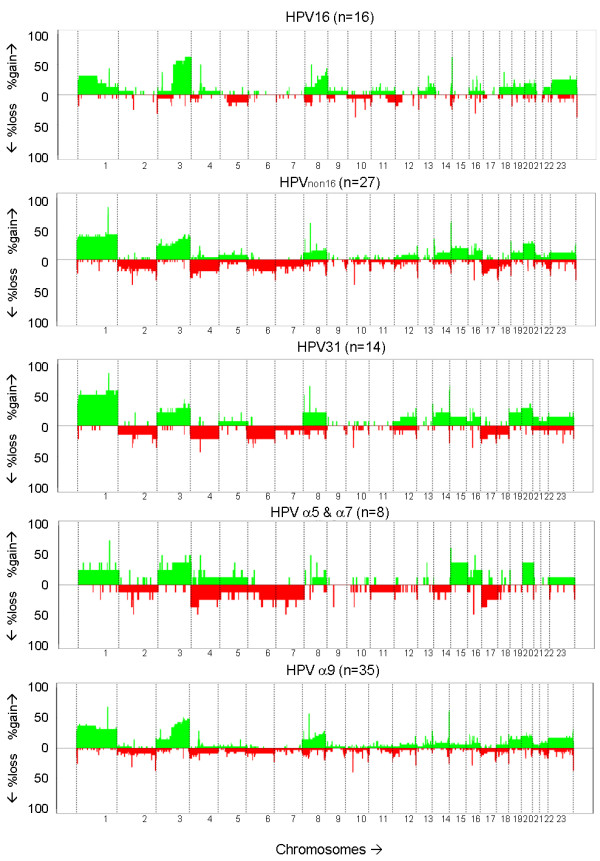
**Frequency plots of the chromosomal aberrations**. Lesions with HPV16 display fewer chromosomal losses than lesions with other hrHPV types or HPV31 in particular.

To determine whether there was a difference in affected regions in CIN2/3 with different hrHPV types as well as different species, the frequency of gains and losses per oligonucleotide was plotted (Figure 3). Aberrations occurring in ≥ 20% of the CIN2/3 cases with HPV16 included gained regions on chromosomes 1p, 3q, 8q and X. For CIN2/3 with HPV_non16 _these included gained regions on 1, 3, 15q, 20 and Xq and lost regions on 2q, 4p, 7q, 12q and 17p, and, for CIN2/3 with HPV31 gained regions on 1, 3, 8q, 14q, 19p, 20 and Xq and lost regions on 2q, 4, 6p, 7q, 8q, 12q and 17. For CIN2/3 with HPVα9, aberrations in ≥ 20% of the cases involved gains on chromosomes 1, 3q, 8q and 20 and losses on 17q. For CIN2/3 with HPVα5&α7 these were gains on 1, 3, 4q, 5q, 15q, 16q and 20 and lost regions on 2, 4, 6q, 7, 14q, 17 and Xp.

We next determined whether specific chromosomal regions were differentially affected between lesions harbouring the various hrHPV types. Comparison of lesions with HPV16 (n = 14) to HPV_non16 _(n = 20) did not reveal regions that were significantly different. However, comparison of lesions with HPV16 (n = 14) to those with HPV31 (n = 10) indicated both lost and gained chromosomal regions that were significantly more affected in lesions harbouring HPV31 (Table 2). These included lost regions on chromosomes 2q, 4p, 4q, 6p, 6q, 8q & 17p and gained regions on 1p & 1q. Comparison of lesions with HPVα9 (n = 28) to HPVα5&α7 (n = 6) did not reveal regions that were significantly different.

**Table 2:** Altered chromosomal regions showing significant differences between lesions with HPV31 compared to those with HPV16 (FDR < 0.2).

		Number of CIN2/3 cases loss vs. no loss				
**Region**	**Cytoband**	**HPV16 loss**	**HPV31 loss**	**FDR**	**HPV16 gain**	**HPV31 gain**

chr2:222405165-227437895	2q36.1-q36.3	0	3	0.123	0	0

chr2:229728860-238929377	2q36.3-q37.3	0	3	0.123	0	0

chr4:49276564-62563622	4p11-q13.1	0	3	0.132	1	0

chr4:71160319-78914719	4q13.3-q21.1	0	3	0.132	1	0

chr4:79281456-84030000	4q21.21-q21.22	0	3	0.132	1	0

chr4:88363994-132792857	4q21.3-q28.3	0	3	0.132	1	0

chr4:133037098-163174205	4q28.3-q32.2	0	3	0.132	1	0

chr4:163304315-191027815	4q32.2-q35.2	0	3	0.132	1	0

chr6:431607-3540777	6p25.3-p25.2	0	3	0.121	1	0

chr6:7902624-26085970	6p24.3-p22.2	0	3	0.121	0	0

chr6:27956910-31362796	6p22.1-p21.33	0	3	0.121	0	0

chr6:32718990-36975126	6p21.32-p21.2	0	3	0.121	0	0

chr6:42755363-48175246	6p21.1-p12.3	0	3	0.121	0	0

chr6:48648135-106496239	6p12.3-q21	0	3	0.121	0	0

chr6:107106382-169915340	6q21-q27	0	3	0.121	0	0

chr8:142600766-145818157	8q24.3	0	3	0.127	5	3

chr17:3564934-7259932	17p13.2-p13.1	0	3	0.122	0	0

chr17:7328200-10781444	17p13.1-p12	0	3	0.122	0	0

chr17:12531028-22078641	17p12-p11.2	0	3	0.122	0	0

		Number of CIN2/3 cases gain vs. no gain				

Region	Cytoband	HPV16gain	HPV31gain	FDR	HPV16loss	HPV31loss

chr1:107415557-114310216	1p13.3-p13.2	3	7	0.163	0	0

chr1:114666834-146812099	1p13.2-q21.2	3	7	0.163	0	0

chr1:148081768-151210846	1q21.2-q21.3	3	7	0.163	0	0

chr1:151380037-167624107	1q21.3-q24.3	3	7	0.163	0	0

chr1:168079451-172935386	1q24.3-q25.2	3	7	0.163	0	0

chr1:172950731-177727312	1q25.2-q25.3	2	7	0.063	0	0

chr1:177742624-194855793	1q25.3-q31.3	2	8	0.029	0	0

chr1:195212008-208655399	1q31.3-q32.3	2	8	0.029	0	0

chr1:212426726-217520338	1q41	2	8	0.029	0	0

chr1:218155880-223642833	1q41-q42.13	2	8	0.029	0	0

chr1:223683098-230121747	1q42.13-q42.2	2	7	0.063	0	0

chr1:231048153-234780716	1q42.3-q43	2	7	0.063	0	0

chr1:236021319-242608344	1q43-q44	2	8	0.029	0	0

chr1:242848259-246751472	1q44	2	8	0.029	0	0

All lesions included in the analysis had > 1.5% aberrations

All known genes within the affected chromosomal regions differing between lesions with HPV16 compared to HPV31 were subjected to Ingenuity Pathway Analysis. The top 3 canonical pathways allocated to these genes involve the antigen presentation pathway, allograft rejection signaling and cytotoxic T-lymphocyte-mediated apoptosis of target cells. The majority of the genes in these pathways overlap and involve major histocompatibility complex molecules located at chromosome 6p, which was found to be more frequently lost in HPV31-positive lesions (see Additional file 1: Table S1).

## Discussion

Chromosomal profiles of CIN2/3 infected with different hrHPV types were compared in order to determine the potential contribution of different hrHPV types to the heterogeneity in chromosomal profiles as observed previously in CIN2/3 [21].

Unsupervised hierarchical clustering analysis revealed considerable heterogeneity between CIN2/3 with respect to their chromosomal aberrations, with one subset having relatively few aberrations (cluster 1) and the other with an increased number of aberrations (cluster 2), similar to our observations in earlier studies [21]. It should be noted that, even though all lesions were associated with long-term hrHPV infections (≥ 5 years), there was still a subset of lesions with few chromosomal aberrations. This is in line with our previous study comparing lesions with < 5 years versus ≥ 5 years preceding hrHPV infection. In addition to the majority of lesions with short-term infection (< 5 years), also a subset of lesions with long-term HPV infection (≥ 5 year) showed rather few chromosomal aberrations as well. Interestingly, the majority of lesions with HPV16 (87.5%) were in the relatively quiet cluster (cluster 1) and had few aberrations. Within this cluster a subgroup could be recognised that consisted primarily of lesions with HPV16 that had gain of 3q and/or 1. The fact that CIN3 with a short-term HPV16 infection, likely reflecting the fast-progressing HPV16 lesions, also showed few chromosomal aberrations [6], indicates that the overall detection of fewer aberrations in HPV16 positive lesions is not dependent on duration of HPV16 infection. It should, however, be noted that only women over 30 years of age were included in the study since they were derived from the POBASCAM trial.

The detection of fewer copy number aberrations in HPV16-positive lesions was furthermore corroborated by hrHPV-typing analysis of an independent set of CIN2/3 previously analysed by arrayCGH [21], which also demonstrated that the majority of CIN2/3 with HPV16 (81.8%) clustered together and showed relatively few aberrations (data not shown).

Related to the clustering results, the average percentage of chromosomal losses in lesions with HPV31 was three-fold higher compared to the HPV16-positive lesions, i.e. 6.7% versus 2.2% (Table 1). No conclusion on the distribution over the two clusters of the remaining lesions harbouring other hrHPV types could be drawn due to their low numbers. However, lesions harbouring α5&α7 types had two-fold more losses compared to α9 positive lesions, i.e. 8.5% versus 4.2% (Table 1).

The observation that lesions with HPV16 tended to contain fewer aberrations than those with different types may indicate that HPV16 causes faster progression than other types and/or may not need as many chromosomal aberrations for progression to CIN2/3. The idea that lesions with HPV16 may progress faster seems to be supported by data from Vinokurova *et al*. [36], who reported a significant difference in the age of cervical cancer patients harbouring different hrHPV types. While the age at CIN3 diagnosis was not significantly different for HPV16-positive women compared to HPV31-positive women, women with an HPV16-positive carcinoma had a median age of 43 years, whereas those with HPV31-containing carcinomas were on average 64 years of age (*p *< 0.01). Also, other studies reported hrHPV type-related differences in that women with HPV16-and HPV18-positive carcinomas were younger than those infected with other types [26,31,37-39].

Comparison of lesions with HPV16 to HPV31 revealed significant differences in the affected chromosomal regions. In particular, losses at chromosomes 2q, 4p, 4q, 6p, 6q, 8q & 17p and gain of 1p & 1q were significantly more frequent in lesions with HPV31. Ingenuity Pathway Analysis indicated that genes located within significantly more frequently altered chromosomal regions in HPV31-positive lesions were particularly involved in pathways related to the immune response, most of which are related to loss of the locus encoding the major histocompatibility complex molecules (human leukocyte antigen, HLA) at chromosome 6p.

While not significant, lesions with HPV16 had the highest incidence of 3q gain, which was almost two-fold higher compared to HPV_non16 _or HPV31. Gain of 3q is one of the most consistent chromosomal aberrations in cervical carcinoma [40-46] and has been suggested to predict progression of CIN [47]. Genes located at 3q, as was recently shown for PIK3CA [48], may have an important role in malignant transformation. It is tempting to speculate that HPV16-positive lesions with this particular aberration may not require many additive copy number aberrations for progression.

It should be noted that by arrayCGH analysis only copy number aberrations can be detected. Hence, it cannot be excluded that lesions with HPV16 have other, potentially more subtle genomic or (epi)genetic aberrations, such as mutations, loss of heterozygosity or DNA methylation. The fact that HPV16E6E7 have convincingly been demonstrated to induce genetic instability in *in vitro *model systems (reviewed by Korzenievski [49]), would argue for the presence of a genetic instable environment in HPV16-positive CIN3. We anticipate that the aberrations detected with arrayCGH result from their selective growth advantage becoming evident upon selection pressure during multiple cell divisions. This is substantiated by a recent paper by Bester *et al*. [50] showing an immediate induction of replication induced DNA damage upon HPV16E6E7 expression, whereas loss of heterozygosity and copy number variations only became evident after 100-250 population doublings.

Immune evasion is important for the persistence of HPV and may be achieved by various mechanisms, including HLA loci being affected by chromosomal loss, loss of heterozygosity, viral integration or mutations, which may result in functional loss [51-58]. Indeed expression of HLA class I has been reported to decrease progressively with cervical lesion grade, [59,60]. Immune evasion may also occur via direct interaction between viral and host proteins. For HPV16, for instance, direct interaction of E5 with the hydrophobic domain of HLA class I heavy chain was found to prevent its transport to the cell surface [61], whereas HPV16 E7 can trigger down-regulation of *HLA class I *gene transcription [62]. However, neither the mechanisms nor efficiency of HLA class I down-regulation has been investigated for HPV31 and as such it is not feasible to perform comparisons to HPV16. It may be that the respective viral proteins of other hrHPV types, such as HPV31, are less capable of interfering with antigen presentation, so that persistence in transforming infections becomes more dependent on (epi)genetic aberrations within the HLA locus. Interestingly, lesions with HPV31 also had significantly more losses at the location of the *p53 *gene on chromosome 17. It is still questionable to what extend p53 activity is affected in these cases. It may be that the E6 protein of HPV16 is more efficient in inactivation of p53 at the posttranslational level than E6 of HPV31 and that for this type additional events are needed to inactivate *p53*. Indeed, the study of Ku *et al*. reported that in cervical carcinomas the majority of cases with *p53 *mutations and LOH at 17p occurred in carcinomas harbouring types other than HPV16 [63]. However, *in vitro *experiments do not provide evidence of differences in degradation efficiency of the E6 proteins of these different hrHPV types [64].

To the best of our knowledge, there are no other studies in which the relation between specific hrHPV types and chromosomal aberrations in precursor lesions has been examined. So far, only a study on cervical carcinomas demonstrated the presence of type-dependent chromosomal aberrations [65]. Carcinomas without detectable HPV or containing HPV18 in a single or multiple infection showed a higher incidence of gains at 20q compared to cases containing HPV16 or other types [65]. Comparison of the frequency of 20q gain in CIN2/3 with HPV16 versus HPV18 was not possible in our data set due to the low numbers of lesions harbouring HPV18. Comparison of the frequency of 20q gain in HPVα9-versus HPVα5&α7-positive lesions, however, indicated an increased frequency in lesions harbouring types from the α5 and α7 species (i.e. 25.8% vs. 36.7%). Gain of 20q was also more frequent in lesions with HPV_non16 _or HPV31 in particular, compared to those with HPV16 (29.6%, 35.7% vs. 25.0% respectively).

## Conclusions

Present data indicate that the chromosomal signatures in CIN2/3 are at least in part related to the hrHPV type present. Lesions with HPV16 displayed fewer chromosomal aberrations than lesions with other hrHPV types. Specific differences, when comparing lesions with HPV16 to HPV31, included losses at chromosome 6p encoding major histocompatibility genes. One could speculate that these type-specific aberrations in lesions with HPV_non16 _types facilitate viral persistence and high-grade CIN development.

## Competing interests

C.J.L.M. Meijer, P.J.F. Snijders and R.D.M. Steenbergen have relationships with Self-screen B.V., a spin-off company of VU university medical center, The Netherlands. All other authors declared no conflict of interest.

## Authors' contributions

MB performed experiments and drafted the manuscript. SMW, CJLMM, PJFS and RDMS participated in the design of the study, interpretation of data and revised the manuscript. WNvW and MAvdW contributed to the array data analysis. BY provided the infrastructure and know-how for the microarray experiments. All authors have read and approved the final manuscript.

## Online supporting information

Array data is available from the Gene Expression Omnibus (GEO, http://www.ncbi.nlm.nih.gov/projects/geo/) through series accession number GSE31241.

## Pre-publication history

The pre-publication history for this paper can be accessed here:

http://www.biomedcentral.com/1471-2407/12/36/prepub

## Supplementary Material

Additional file 1 **Table S1**. Genes within the top 3 canonical pathways. Click here for file
