# Supplementary material for: HPV type-related chromosomal profiles in high-grade cervical intraepithelial neoplasia
Source: BMC Cancer. 2012 Jan 24;12:36. doi: 10.1186/1471-2407-12-36 (PMC3305644; doi:10.1186/1471-2407-12-36)
Supplement: Additional file 1 — Table S1. Genes within the top 3 canonical pathways. [file 1471-2407-12-36-S1.DOC]

**Supplementary** **Table I:** Genes within the top 3 canonical pathways.

| **Antigen Presentation Pathway** | | | | |
| --- | --- | --- | --- | --- |
| GenBank | Entrez Gene Name | Location | Type(s) | Cytoband |
| *HLA-A* | major histocompatibility complex, class I, A | Plasma Membrane | transmembrane receptor | 6p21.33 |
| *HLA-C* | major histocompatibility complex, class I, C | Plasma Membrane | transmembrane receptor | 6p21.33 |
| *HLA-DMA* | major histocompatibility complex, class II, DM alpha | Plasma Membrane | transmembrane receptor | 6p21.32 |
| *HLA-DMB* | major histocompatibility complex, class II, DM beta | Plasma Membrane | transmembrane receptor | 6p21.32 |
| *HLA-DOA* | major histocompatibility complex, class II, DO alpha | Plasma Membrane | transmembrane receptor | 6p21.32 |
| *HLA-DOB* | major histocompatibility complex, class II, DO beta | Plasma Membrane | transmembrane receptor | 6p21.32 |
| *HLA-DPA1* | major histocompatibility complex, class II, DP alpha 1 | Plasma Membrane | transmembrane receptor | 6p21.32 |
| *HLA-DPB1* | major histocompatibility complex, class II, DP beta 1 | Plasma Membrane | transmembrane receptor | 6p21.32 |
| *HLA-DQA1* | major histocompatibility complex, class II, DQ alpha 1 | Plasma Membrane | transmembrane receptor | 6p21.32 |
| *HLA-DQA2* | major histocompatibility complex, class II, DQ alpha 2 | Plasma Membrane | transmembrane receptor | 6p21.32 |
| *HLA-DQB2* | major histocompatibility complex, class II, DQ beta 2 | Plasma Membrane | transmembrane receptor | 6p21.32 |
| *HLA-E* | major histocompatibility complex, class I, E | Plasma Membrane | transmembrane receptor | 6p21.33 |
| *HLA-F* | major histocompatibility complex, class I, F | Plasma Membrane | transmembrane receptor | 6p22.1 |
| *HLA-G* | major histocompatibility complex, class I, G | Plasma Membrane | transmembrane receptor | 6p21.33 |
| *HLA-L* | major histocompatibility complex, class I, L, pseudogene | unknown | other | 6p21.33 |
| *PSMB6* | proteasome (prosome, macropain) subunit, beta type, 6 | Cytoplasm | peptidase | 17p13.2 |
| *PSMB8* | proteasome (prosome, macropain) subunit, beta type, 8 (large multifunctional peptidase 7) | Cytoplasm | peptidase | 6p21.32 |
| *PSMB9* | proteasome (prosome, macropain) subunit, beta type, 9 (large multifunctional peptidase 2) | Cytoplasm | peptidase | 6p21.32 |
| *TAP1* | transporter 1, ATP-binding cassette, sub-family B (MDR/TAP) | Cytoplasm | transporter | 6p21.32 |
| *TAP2* | transporter 2, ATP-binding cassette, sub-family B (MDR/TAP) | Cytoplasm | transporter | 6p21.32 |
| *TAPBP* | TAP binding protein (tapasin) | Cytoplasm | transporter | 6p21.32 |
| *MR1* | major histocompatibility complex, class I-related | Plasma Membrane | transmembrane receptor | 1q25.3 |

| **Allograft Rejection Signaling** | | | | |
| --- | --- | --- | --- | --- |
| GenBank | Entrez Gene Name | Location | Type(s) | Cytoband |
| *HLA-A* | major histocompatibility complex, class I, A | Plasma Membrane | transmembrane receptor | 6p21.33 |
| *HLA-C* | major histocompatibility complex, class I, C | Plasma Membrane | transmembrane receptor | 6p21.33 |
| *HLA-DMA* | major histocompatibility complex, class II, DM alpha | Plasma Membrane | transmembrane receptor | 6p21.32 |
| *HLA-DMB* | major histocompatibility complex, class II, DM beta | Plasma Membrane | transmembrane receptor | 6p21.32 |
| *HLA-DOA* | major histocompatibility complex, class II, DO alpha | Plasma Membrane | transmembrane receptor | 6p21.32 |
| *HLA-DOB* | major histocompatibility complex, class II, DO beta | Plasma Membrane | transmembrane receptor | 6p21.32 |
| *HLA-DPA1* | major histocompatibility complex, class II, DP alpha 1 | Plasma Membrane | transmembrane receptor | 6p21.32 |
| *HLA-DPB1* | major histocompatibility complex, class II, DP beta 1 | Plasma Membrane | transmembrane receptor | 6p21.32 |
| *HLA-DQA1* | major histocompatibility complex, class II, DQ alpha 1 | Plasma Membrane | transmembrane receptor | 6p21.32 |
| *HLA-DQA2* | major histocompatibility complex, class II, DQ alpha 2 | Plasma Membrane | transmembrane receptor | 6p21.32 |
| *HLA-DQB1* | major histocompatibility complex, class II, DQ beta 1 | Plasma Membrane | transmembrane receptor | 6p21.32 |
| *HLA-DQB2* | major histocompatibility complex, class II, DQ beta 2 | Plasma Membrane | transmembrane receptor | 6p21.32 |
| *HLA-E* | major histocompatibility complex, class I, E | Plasma Membrane | transmembrane receptor | 6p21.33 |
| *HLA-F* | major histocompatibility complex, class I, F | Plasma Membrane | transmembrane receptor | 6p22.1 |
| *HLA-G* | major histocompatibility complex, class I, G | Plasma Membrane | transmembrane receptor | 6p21.33 |
| *IL2* | interleukin 2 | Extracellular Space | cytokine | 4q27 |
| *FASLG* | Fas ligand (TNF superfamily, member 6) | Extracellular Space | cytokine | 1q24.3 |
| *FCER1G* | Fc fragment of IgE, high affinity I, receptor for; gamma polypeptide | Plasma Membrane | transmembrane receptor | 1q23.3 |
| *IL10* | interleukin 10 | Extracellular Space | cytokine | 1q32.1 |

| **Cytotoxic T Lymphocyte-mediated Apoptosis of Target Cells** | | | | |
| --- | --- | --- | --- | --- |
| GenBank | Entrez Gene Name | Location | Type(s) | Cytoband |
| *CASP3* | caspase 3, apoptosis-related cysteine peptidase | Cytoplasm | peptidase | 4q35.1 |
| *CASP6* | caspase 6, apoptosis-related cysteine peptidase | Cytoplasm | peptidase | 4q25 |
| *HLA-A* | major histocompatibility complex, class I, A | Plasma Membrane | transmembrane receptor | 6p21.33 |
| *HLA-C* | major histocompatibility complex, class I, C | Plasma Membrane | transmembrane receptor | 6p21.33 |
| *HLA-DMA* | major histocompatibility complex, class II, DM alpha | Plasma Membrane | transmembrane receptor | 6p21.32 |
| *HLA-DMB* | major histocompatibility complex, class II, DM beta | Plasma Membrane | transmembrane receptor | 6p21.32 |
| *HLA-DOA* | major histocompatibility complex, class II, DO alpha | Plasma Membrane | transmembrane receptor | 6p21.32 |
| *HLA-DOB* | major histocompatibility complex, class II, DO beta | Plasma Membrane | transmembrane receptor | 6p21.32 |
| *HLA-DPA1* | major histocompatibility complex, class II, DP alpha 1 | Plasma Membrane | transmembrane receptor | 6p21.32 |
| *HLA-DPB1* | major histocompatibility complex, class II, DP beta 1 | Plasma Membrane | transmembrane receptor | 6p21.32 |
| *HLA-DQA1* | major histocompatibility complex, class II, DQ alpha 1 | Plasma Membrane | transmembrane receptor | 6p21.32 |
| *HLA-DQA2* | major histocompatibility complex, class II, DQ alpha 2 | Plasma Membrane | transmembrane receptor | 6p21.32 |
| *HLA-DQB1* | major histocompatibility complex, class II, DQ beta 1 | Plasma Membrane | transmembrane receptor | 6p21.32 |
| *HLA-DQB2* | major histocompatibility complex, class II, DQ beta 2 | Plasma Membrane | transmembrane receptor | 6p21.32 |
| *HLA-E* | major histocompatibility complex, class I, E | Plasma Membrane | transmembrane receptor | 6p21.33 |
| *HLA-F* | major histocompatibility complex, class I, F | Plasma Membrane | transmembrane receptor | 6p22.1 |
| *HLA-G* | major histocompatibility complex, class I, G | Plasma Membrane | transmembrane receptor | 6p21.33 |
| *CD247* | CD247 molecule | Plasma Membrane | transmembrane receptor | 1q24.2 |
| *FASLG* | Fas ligand (TNF superfamily, member 6) | Extracellular Space | cytokine | 1q24.3 |
| *FCER1G* | Fc fragment of IgE, high affinity I, receptor for; gamma polypeptide | Plasma Membrane | transmembrane receptor | 1q23.3 |
